# Post - prandial rise of microvesicles in peripheral blood of healthy human donors

**DOI:** 10.1186/1476-511X-10-47

**Published:** 2011-03-21

**Authors:** Vid Šuštar, Apolonija Bedina-Zavec, Roman Štukelj, Mojca Frank, Eva Ogorevc, Rado Janša, Keriya Mam, Peter Veranič, Veronika Kralj-Iglič

**Affiliations:** 1 Laboratory of Clinical Biophysics, Faculty of Medicine, University of Ljubljana, Lipičeva ulica 2, Ljubljana, Slovenia; 2 Laboratory of Biosynthesis and Biotransformation, National Institute of Chemistry, Hajdrihova ulica 19, Ljubljana, Slovenia; 3 Department of Rheumatology, University Medical Centre Ljubljana, Vodnikova cesta 62, Ljubljana, Slovenia; 4 Laboratory of Biophysics, Faculty of Electrical Engineering, University of Ljubljana, Tržaška cesta 25, Ljubljana, Slovenia; 5 Department of Gastroenterology, University Medical Centre Ljubljana, Japljeva ulica 2, Ljubljana, Slovenia; 6 FEI Company, Europe NanoPort, Achtseweg Noord 5, Bldg, 5651 GG Eindhoven, The Netherlands; 7 Institute of Cell Biology, Faculty of Medicine, University of Ljubljana, Lipičeva ulica 2, Ljubljana, Slovenia

## Abstract

**Background:**

Microvesicles isolated from body fluids are membrane - enclosed fragments of cell interior which carry information on the status of the organism. It is yet unclear how metabolism affects the number and composition of microvesicles in isolates from the peripheral blood.

**Aim:**

To study the post - prandial effect on microvesicles in isolates from the peripheral blood of 21 healthy donors, in relation to blood cholesterol and blood glucose concentrations.

**Results:**

The average number of microvesicles in the isolates increased 5 hours post - prandially by 52%; the increase was statistically significant (p = 0.01) with the power P = 0.68, while the average total blood cholesterol concentration, average low density lipoprotein cholesterol concentration (LDL-C) and average high density lipoprotein cholesterol concentration (HDL-C) all remained within 2% of their fasting values. We found an 11% increase in triglycerides (p = 0.12) and a 6% decrease in blood glucose (p < 0.01, P = 0.74). The post - prandial number of microvesicles negatively correlated with the post - fasting total cholesterol concentration (r = - 0.46, p = 0.035) while the difference in the number of microvesicles in the isolates between post - prandial and post - fasting states negatively correlated with the respective difference in blood glucose concentration (r = - 0.39, p = 0.05).

**Conclusions:**

In a population of healthy human subjects the number of microvesicles in isolates from peripheral blood increased in the post - prandial state. The increase in the number of microvesicles was affected by the fasting concentration of cholesterol and correlated with the decrease in blood glucose.

## Background

In the final stage of the complex process of membrane budding [1-4], buds are pinched off from the cell membrane to become microvesicles (MVs). These submicron - sized membrane - enclosed fragments of cell interior [5,6], which contain biologically important molecules (such as nucleic acids and proteins), reflect the composition of the mother cell [7,8]. They can move with body fluids and are able to reach distal cells. Upon interaction of MVs with distal cells, biologically important molecules from the mother cell may induce activity in the host cell. Thereby MVs represent a cell - to - cell communication mechanism within the body. MVs may carry organelles and viruses [8] and are involved in spreading inflammation [9], in autoimmune diseases [10] and in tumour progression [11]. Further, MVs in blood are associated with tissue factor [12] and are considered prothrombogenic [13].

It has been indicated that cholesterol plays an important role in budding and vesiculation, especially due to partitioning into cholesterol-enriched membrane rafts [14-16] which favour strongly curved membrane regions [17,18]. Due to the self-consistent minimization of the free energy and lateral distribution of membrane constituents, a highly curved local membrane shape with an increased content of constituents that favour such curvature is attained and budding of the membrane is promoted [19-23]. This suggests that membrane budding and vesiculation is connected to the content and redistribution of cholesterol in the membrane, which in turn depends on blood levels of cholesterol and also on cholesterol metabolism. In an in vitro study it was found that the cholesterol concentration in the surrounding medium and in the membrane considerably affects microvesiculation of the membranes of epithelial cells [24] which is also supported by previous findings that membrane rafts (being enriched in cholesterol) are precursors of MVs [23]. We hypothesized that intake of cholesterol will at certain times cause enrichment of membranes with cholesterol, induce formation of membrane rafts, promote membrane budding and lead to increased microvesiculation. Previous studies involving MVs in a healthy human population revealed a post-prandial increase of endothelial - derived [25,26] and plasma total MVs [27,28] in isolates from peripheral blood, while studies on lipoprotein and glucose metabolism report on time - dependences of post-prandial levels of blood cholesterol and glucose [25-31]. It is however unclear what are the mechanisms of interaction of blood cholesterol with membrane cholesterol and with membrane budding and vesiculation. In order to elucidate these mechanisms, it was the aim of the present study to determine the post-prandial effect on the number of MVs in isolates from peripheral blood, in relation to blood cholesterol and blood glucose concentrations.

## Results

### Subjects

Data on subjects' gender, age, MVs, cholesterol and glucose are presented in Table 1. For final analysis we included 21 subjects, 10 females (average age 29 years ± standard deviation 13 years) and 11 males (31 years ± 10 years). In most subjects the glucose concentration decreased post - prandially with concomitant increase in triglycerides, HDL-C and MVs and with a slight decrease in LDL-C. However, in 5 subjects (2 male and 3 female), triglycerides decreased while in one subject (#15, Table 1), all the parameters considered decreased 5 hours after the meal.

**Table 1:** Data on subjects' gender (M: male, F: female), age, the number of microvesicles (MVs), concentrations of total cholesterol (Chol), blood glucose (Glu), triglycerides (Tgl), HDL-C and LDL-C, in the post - fasting (pfast) and post - prandial (pprand) states.

Subject (gender)	Age [years]	MVs pfast	MVs pprand	Chol pfast [mmol/L]	Chol pprand [mmol/L]	Glu pfast [mmol/L]	Glu pprand [mmol/L]	Tgl pfast [mmol/L]	Tgl pprand [mmol/L]	**HDL**-**C pfast [mmol/L]**	**HDL**-**C pprand [mmol/L]**	LDL-C Pfast [mmol/L]	LDL-C Pprand [mmol/L]
**1 **(F)	44	0.51	1.40										

**2 **(M)	51	0.52	0.54	5.9	5.9								

**3 **(F)	20	0.78	1.70	4	3.8	4.7	3.7	1	1	1.5	1.7	1.9	1.7

4 (M)	32	0.25	0.32	4.5	4.7	5.4	5.1	0.7	1.1	1.5	1.5	2.5	2.5

**5 **(F)	20	0.16	0.80	4.8	4.6	4.8	3.2	0.8	1.4	2	2	2.2	2

**6 **(F)	47	0.60	0.35	5.9	6	5.4	4.8	0.7	0.8	1.6	1.6	3.8	3.8

**7 **(M)	42	0.14	0.32	7.4	7.1	5.1	4.7	1.2	1.2	1.8	1.9	5	4.8

**8 **(F)	20	0.23	0.32	5.4	5.5	4.9	4.6	0.9	0.7	1.8	1.8	3.2	3.3

**9 **(M)	20	0.41	0.46	4.3	4.1	4.8	5	0.7	1	1.5	1.7	2.2	2.1

**10 **(M)	22	0.36	0.52	5.0	4.8	5	4.9	0.8	0.8	1.5	1.5	2.9	2.8

**11 **(F)	51	0.84	1.25	5.2	5.4	5.6	5.5	0.7	1.7	1.5	1.5	3.2	3.1

**12 **(F)	20	0.57	2.70	3.8	3.8	4.7	5.0	1.0	0.9	1.2	1.3	2.0	2.1

**13 **(M)	29	0.30	0.23	5.2	5.2	4.5	4.8	0.8	0.8	1.5	1.5	3.3	3.2

**14 **(M)	27	0.22	0.27	4.8	4.9	5.1	5.2	1	0.4	1.5	1.3	2.9	3

**15 **(M)	22	1.11	0.80	4.8	4.7	5.3	4.8	1.3	0.9	1.5	1.5	2.8	2.7

**16 **(M)	43	0.74	1.48	5.5	5.4	4.8	4.7	1.3	2	1.4	1.4	3.4	3.3

**17 **(F)	27	0.93	0.58	5.5	5.0	4.5	4.8	1	1.1	1.9	1.6	3	2.7

**18 **(M)	31	0.24	0.71	3.3	3.5	5.7	4.8	0.8	0.9	1.7	1.6	1.4	1.5

**19 **(F)	20	1.16	1.06	4.2	4.4	5.1	4.6	0.9	0.7	1.5	1.6	2.3	2.3

**20 **(F)	20	0.49	2.52	4.3	4.8	5	4.7	1.1	1.2	1.4	1.6	2.4	2.5

**21 **(M)	20	0.52	0.60	5.3	5.6	5	4.6	1.4	1.5	1.3	1.4	3.5	3.5

**mean**	30	0.53	0.90	4.96	5.05	5.02	4.71	0.95	1.06	1.56	1.58	2.84	2.78

**SD**	11	0.30	0.71	0.90	0.93	0.34	0.49	0.22	0.38	0.20	0.18	0.81	0.79

None of the parameters measured post - fasting differed statistically significantly between women and men. Averaged over the post - fasting and post - prandial results, MVs were more abundant in the female population (average 0.95 ± standard deviation 0.70) than in the male population (0.50 ± 0.32). Τhe male population had slightly higher concentrations of total cholesterol ((5.09 ± 0.96) mmol/L), glucose ((4.97 ± 0.29) mmol/L), triglycerides ((1.03 ± 0.35) mmol/L) and LDL-C ((2.97 ± 0.89) mmol/L) than the female population where the concentration of total cholesterol was ((4.80 ± 0.73) mmol/L), of glucose ((4.76 ± 0.57) mmol/L), of triglycerides ((0.98 ± 0.26) mmol/L) and of LDL-C ((2.64 ± 0.65) mmol/L). The concentration of HDL-C was higher in the female population ((1.62 ± 0.22) mmol/L) than in the male population ((1.52 ± 0.15) mmol/L). The two populations differed statistically significantly only in the concentration of MVs (p = 0.03) while differences in all other parameters were statistically insignificant.

### Determination of MVs in isolates

**Figure 1:**
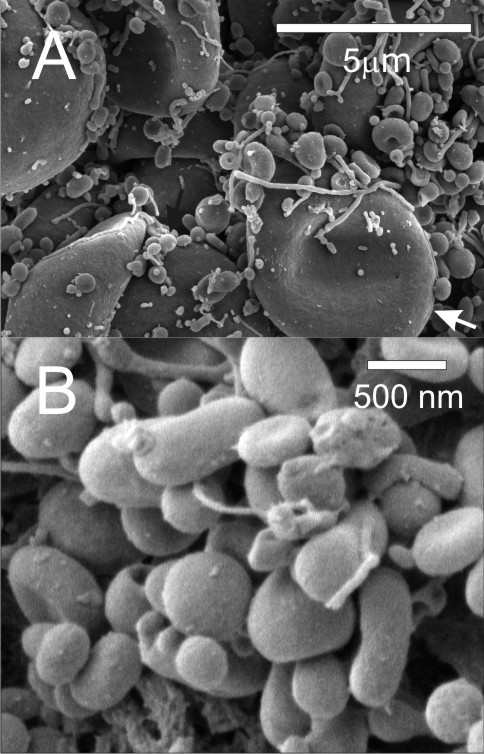
**Scanning electron micrograph of an isolate from peripheral blood of a healthy human subject**. A: numerous microvesicles (black arrow) and residual cells (white arrow), B: high magnification image of microvesicles.

**Figure 2:**
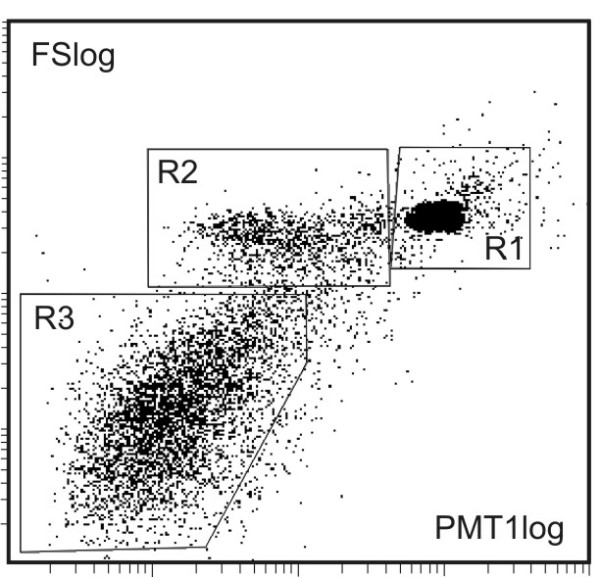
**A typical dot plot detected by the flow cytometer**. FS: forward scattered light, PMT1: side scattered light. The three regions marked estimate R1: microspheres with 10 μm diameter, R2: residual cells and R3: microvesicles.

**Figure 3:**
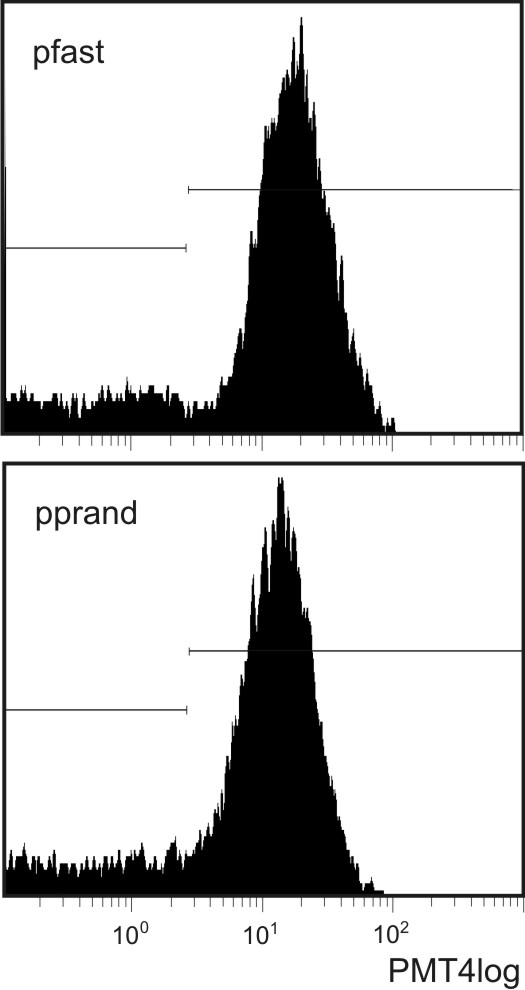
**Flow cytometry histogram showing the intensity of fluorescent light emitted by microvesicles in the post - fasting and in the post - prandial states**. PMT4 detects light expected from anti-CD42b-PE molecules.

Imaging of isolated material (Figure 1) shows MVs (A, B) and residual cells (A). MVs were mostly globular. Their effective diameter (averaged over 85 MVs) was 394 nm (SD = 7.4 nm). Figure 2 shows a dot plot of events detected by the flow cytometer. As cells are expected to be found in isolates (Figure 1), we defined three regions; R1: microspheres with known diameter 10 μm, R2: residual cells, and R3: MVs. Labelling of isolates with antibodies which interact with endothelial, platelet and erythrocyte surface molecules (CD31/CD42b and CD235, respectively) showed no difference in the distribution of MVs with respect to the origin between the post - fasting and the post - prandial states (Table 2). In labelling with anti-CD31-FITC and anti-CD42b-PE, 27%/30% of events (in the post - fasting/post - prandial state) corresponded to unlabelled particles. Out of these, 18%/15% can be ascribed to anti-CD235-FITC labelled particles and 4.5%/9% to the background. This leaves around 6% of unlabelled particles in the isolates in the post - fasting and in the post - prandial state. Figure 3 shows the distribution of the intensity of the emitted fluorescent light expected from anti-CD42b-PE positive MVs in the post - fasting and in the post - prandial states. No significant difference can be observed between the two distributions.

**Table 2:** Percent of fluorescent light - emitting particles in isolates from peripheral blood of a healthy subject in the post - fasting and in the post - prandial states.

antibody type	percent of fluorescent light - emitting particles	
	**post - fasting**	**post - prandial**

**anti-CD42bPE+ and anti-CD31FITC+**	73%	70%

**anti-CD235FITC+**	18%	15%

**anti-CD31FITC+ and anti-CD42bPE-**	6%	6%

**anti-CD42bPE- and anti-CD31FITC-**	21%	24%

**background**	4,5%	9%

Antibodies interact with platelet (CD42b), erythrocyte (CD235) or platelet/endothelial (CD31) surface molecules: anti-CD31-FITC and anti-CD42-PE positive events correspond to platelet - derived MVs, anti-CD235-FITC positive events correspond to erythrocyte - derived MVs, anti-CD31-FITC positive and anti-CD42b-PE negative events correspond to endothelial - derived MVs and double negative events with respect to anti-CD42b-PE and anti-CD31-FITC correspond to unstained particles in isolates consisting of background, membranous structures for which staining was unsuccessful and sample - derived non - membranous structures.

### MV, cholesterol and glucose profile of the population

The average number of MVs in the isolates from blood was considerably (52%) and statistically significantly higher (p < 0.01) in the post - prandial than in the post - fasting state (Table 3) with the power P = 0.68 at α = 0.05. The concentrations of total blood cholesterol, LDL-C and HDL-C remained on the average within 2% of the initial (post - fasting) values (Table 3). We found an 11% post - prandial increase in triglycerides concentration (p = 0.12). Also we found a 6% decrease in the blood glucose concentration (p < 0.01) with the power P = 0.74 at α = 0.05 (Table 3). Considering all data (from the first day and from the second day), we obtained a statistically significant positive correlation between the number of MVs and triglycerides concentration (r = 0.31, p = 0.03) and a statistically significant negative correlation between the number of MVs and LDL-C concentration (r = - 0.27, p = 0.05), (Table 4).

**Table 3:** Differences between post-prandial and post-fasting states

	average differences between post - prandial and post - fasting States	p (t-test)	P
**MVs**	0.37 (52%)	0.01*	0.68

**Chol**	0.09 mmo/L (2%)	0.93	

**Glu**	-0.31 mmol/L (-6%)	0.01*	0.74

**Tgl**	0.11 mmol/L (11%)	0.12	

**HDL-C**	0.02 mmol/L (1%)	0.24	

**LDL-C**	-0.06 mmol/L (-2%)	0.08	

Differences between the average values of the number of microvesicles between the post -prandial and the post - fasting states, and the respective differences between the average concentrations of total cholesterol (Chol), glucose (Glu), triglycerides (Tgl), HDL-C and LDL-C, the corresponding percentages with respect to the average values (in brackets), statistical significance of the differences (p) and the corresponding statistical power (P). Statistically significant correlations (p < 0.05) are marked by asterisks.

**Table 4:** Correlations between different blood parameters

	MVs	Chol	Glu	Tgl	HDL-C	LDL-C
**MVs**	1	-0.20 (0.11)	-0.18 (0.14)	0.31 (0.03) *	-0.19 (0.12)	-0.27 (0.05) *

**Chol**		1	0.04 (0.39)	0.24 (0.07)	0.27 (0.01) *	0.97 (< 0.001)*

**Glu**			1	-0.19 (0.12)	-0.36 (0.01)*	0.16 (0.17)

**Tgl**				1	-0.56 (0.037) *	0.20 (0.11)

**HDL-C**					1	0.09 (0.30)

Correlations between values of different blood parameters taking into account data from both post - fasting and post - prandial states: the number of microvesicles (MVs), the concentrations of total cholesterol (Chol), glucose (Glu), triglycerides (Tgl), HDL-C and LDL-C. The statistical significance of the correlations is shown in brackets. Statistically significant values (p < 0.05) are marked by asterisks.

Correlations between all the variables considered in the post - fasting and in the post - prandial state are given in Table 5. The upper right triangle presents the Pearson coefficients and the statistical significance of the correlations, while the lower left triangle presents the corresponding slopes of the linear dependences for the correlations that proved statistically significant. The post - prandial number of MVs negatively correlated with the post - fasting total cholesterol concentration (r = - 0.46, p = 0.035). The correlation between the differences in MVs and in glucose concentration was negative and statistically significant (r = - 0.39, p = 0.05).

**Table 5:** Correlations between different post - prandial and post - fasting blood parameters

	MV pfast	Chol pfast	Glu pfast	Tgl pfast	HDL-C pfast	LDL-C pfast	Age	MV ppran	Chol ppran	Glu ppran	Tgl ppran	HDL-C ppran	LDL-C ppran
MV pfast	1	-0.144 (0.535)	-0.013 (0.956)	0.115 (0.630)	-0.225 (0.341)	-0.120 (0.614)	0.015 (0.949)	0.331 (0.132)	-0.155 (0.490)	0.086 (0.711)	0.062 (0.794)	-0.257 (0.274)	-0.157 (0.508)

Chol Pfast		1	-0.059 (0.804)	0.401 (0.080)	0.182 (0.442)	0.974** (< 0.01)	0.480** (0.028)	-0.461* (0.035)	0.965* (< 0.01)	0.088 (0.706)	0.383 (0.096)	0.181 (0.446)	0.939* (< 0.01)

Glu pfast			1	-0.089 (0.710)	-0.045 (0.851)	0.006 (0.979)	0.449* (0.047)	-0.169 (0.477)	0.069 (0.771)	0.380 (0.098)	0.042 (0.859)	-0.090 (0.704)	0.061 (0.799)

Tgl pfast				1	-0.446* (0.049)	0.431 (0.058)	-0.231 (0.328)	0.073 (0.760)	0.347 (0.134)	-0.041 (0.865)	0.693*** (< 0.01)	-0.301 (0.197)	0.392 (0.088)

HDL-C pfast				-0.07	1	0.007 (0.976)	0.143 (0.547)	-0.415 (0.069)	0.116 (0.626)	-0.416 (0.068)	-0.306 (0.189)	0.791** (< 0.01)	-0.029 (0.902)

LDL-C pfast		-0.05				1	0.462** (0.040)	-0.418 (0.067)	0.977** (< 0.01)	0.219 (0.353)	0.360 (0.118)	0.016 (0.946)	0.988** (< 0.01)

Age		-0.001 years×L mmol	0.008 years × L mmol			0.003 years × L/mmol	1	-0.134 (0.553)	0.588** (0.004)	0.360 (0.109)	0.162 (0.494)	-0.031 (0.896)	0.475** (0.034)

MV ppran		0.161 L/mmol						1	-0.301 (0.173)	-0.111 (0.632)	0.129 (0.589)	-0.203 (0.391)	-0.400 (0.081)

Chol ppran		-0.06				0.029	0.959 mmol/L × years		1	0.164 (0.479)	0.337 (0.146)	0.129 (0.588)	0.976** (< 0.01)

Glu ppran										1	-0.058 (0.809)	-0.629* (0.003)	0.282 (0.228)

Tgl ppran				0.027							1	-0.118 (0.619)	0.290 (0.214)

HDL-C ppran					-0.58					0.597		1	-0.029 (0.902)

LDL-C ppran		-0.07				-0.01	1.035 mmol/L × years		-0.36				1

The upper right triangular region (above the diagonal): the Pearson coefficients of correlations between different variables corresponding to the post - fasting (pfast) and to the post - prandial (ppran) states with the statistical significances of correlations given in brackets; the lower left triangular region (below the diagonal): the slopes of statistically significant correlations obtained by assuming a linear dependence of the independent variable given in the first column and the dependent variable given in the first row. Variables give the number of microvesicles 28 (MVs), the concentrations of total cholesterol (Chol), glucose (Glu), triglycerides (Tgl), HDL-C and LDL-C and the age of the donors. Statistically significant correlations (p < 0.05) are marked by asterisks.

## Discussion

We hypothesized that MVs are a possible pool of blood cholesterol, so it was of interest to focus on the potential correlations of the total number of MVs with concentrations of blood cholesterol post - fasting and post - prandial. We believed that the total number of MVs is relevant since all MVs contain cholesterol.

We obtained a considerable (52%), statistically significant increase in the number of MVs after a cholesterol, fat and carbohydrate - rich meal within a population of subjects with no record of disease. The number of MVs increased in 16 and decreased in 5 subjects 5 hours after the meal (Table 1), where the greatest increase was 135% and the greatest decrease 52%. The increase in the total MV concentration (52%) agrees with the result of Michelsen et al. [28] (~ 45%, SD ~ 20%) and of Tushuizen et al. [27] (up to 40%, SD ~ 10%) for healthy controls. These studies included similar numbers of healthy subjects as our study (20 and 17, respectively). In agreement with Michelsen et al. [28], we found a positive correlation between the differences in MVs between the post - prandial and post - fasting states and the respective differences in triglycerides; however, in our sample it was not statistically significant. We found a statistically significant negative correlation between the differences in the number of MVs and the differences in the blood glucose concentration.

Although the average values of parameters, especially of total cholesterol, LDL-C and HDL-C, were only moderately affected by food intake (Table 5), there were large variations of the respective parameters within the population (Table 1) which was observed already by Cohn et al. [29]. Even if the processes were the same in all participants, the dynamics of the blood cholesterol and glucose might vary between individuals, which could cause large variation in data taken at a given time. Measured at a given time after food intake, it therefore cannot be concluded that the post - prandial increase or decrease of any of the parameters is an indicator of a normal or a pathological process at the individual level. Further, based on the existing evidence it cannot be concluded that any particular mechanism keeps the average values of these parameters relatively constant through fasting and post - prandial states. In our sample the post - prandial increase in triglycerides concentration was 11% which is smaller than the peak value reported by other authors [26 - 31], however, as we have measured blood parameters only at a chosen time after the meal we cannot say when the time dependence of triglycerides concentration reached its maximum in our experiment.

We assumed that the post - prandial effect on MVs would be the same in men and women, i.e. the number of MVs would increase after meals in both, however, there could be a difference in the amount of vesiculation due to compositional differences of the cell membrane. If the post - prandial effects differed in magnitude between the sexes, considering both groups together would only increase variance in the statistical analysis. This means that it would be possible that we could get a lower or no statistical significance of the effect in the combined group due to the larger scattering of data. Since the number of MVs increased in women and in men (albeit not to the same magnitude) and statistical significance of the increase was actually shown in the combined group (considering both sexes together), we think our point is made. Adding new data obtained under changed external parameters could be a source of larger error than considering men and women together. Therefore regarding the post - prandial rise of the total number of MVs, we believe that considering both women and men in this study was optimal, albeit not ideal solution within the existing possibilities. It would, however, be of interest to study larger populations of women and men separately.

Our protocol did not restrain the participants from their usual activities and eating habits, nevertheless, the results obtained essentially agree with previous studies in which activities were better defined and controlled [25-28]. On the other hand, our protocol represents better the real life situation of individuals, which is an advantage with respect to other studies.

In interpreting the results, it should be considered that circadian rhythm has an effect on the level of blood constituents. Since on the first day blood was taken at 7 a.m. and on the second day at 12 p.m., the concentrations of blood cholesterol and glucose could differ due to circadian rhythm. Based on the reported dependences of blood cholesterol and triglyceride concentrations on time of day [32,33], we estimated that due to the circadian rhythm effect on average the concentration of total cholesterol would increase by about 3%, the concentration of triglycerides would increase by about 7%, the concentration of LDL-C would decrease by about 2% while the concentration of HDL-C would be unchanged. If these estimates are compared to our results (Table 2), the observed effect on total cholesterol concentration, LDL-C and HDL-C could be ascribed to circadian rhythm, while the effect on the triglycerides is somewhat larger (by 4%). However, subjects considered in the circadian rhythm study received regular meals [32,33].

It is still unclear to what extent MVs found in isolates are actually present in blood at sampling and to what extent the measured MVs are primarily a reflection of the elastic properties of blood cell membranes. The post - prandial change in cholesterol distribution includes alteration of the blood cell membranes [34,35], which in turn affects microvesiculation. However, differences in the ability of cells from different donors or at different cholesterol levels to vesiculate are probably similar if monitored before, during and directly after MVs isolation.

By labelling with antibodies, we could not prove post - prandial redistribution of MVs with respect to origin. However, the variance in the portion of platelet - derived MVs obtained with the same sample of blood in the post - fasting state was 15% (not shown). Matching of the distributions of MVs with respect to origin in both states was excellent (Table 2 Figure 3) in spite of a rather large error expected within the method. However, since such situation was obtained, the comparison between the respective fluorescent light distributions in the post - fasting and the post - prandial states was highly relevant. We observed no significant difference between the two populations of MVs (Figure 3).

Immune complexes could be a possible source of artefact as their sizes overlap with sizes of MVs [36]. However, it was suggested that blood plasma samples of patients with osteoarthritis and rheumatoid arthritis (in which the immune complexes were found elevated) showed only minor antibody staining for immune complexes [36] while in healthy subjects this effect is expected to be even smaller. Further, Figure 1 reveals smooth symmetrical shapes which are characteristic for closed shapes with no internal structures corresponding to MVs.

The estimated portion of platelet - derived MVs was around 70% which is somewhat lower than reported previously (80% [37] and 88-98% [27]), the portion of erythrocyte - derived MVs was around 16% which is higher than reported previously (8% [37], 2-11% [27]) and the portion of endothelial - derived MVs was estimated 6% which is higher than reported previously (~2% [37], 0.8% [26]). Tushuizen et al. [27] found no endothelial - derived MVs in their population of healthy subjects. However, endothelial - derived MVs were not directly determined in our work.

General questions regarding the method of isolation must be addressed in the future in order to improve and standardize the protocol for isolation and determination of MVs. However, information from population studies are also essential and we think that population studies should be performed in the optimum manner, keeping in mind the limitations of the method and avoiding situations where it could not give useful results. A larger pool of data on MVs from different independent groups should be gathered in order to find answers to the role of lipid metabolism in microvesiculation. We believe that it is best that in the present state of the art the experiments within a study be performed within the same set - up. This unfortunately limits the studies to relatively small populations.

## Conclusions

The number of MVs in isolates from peripheral blood increases post - prandially in a population of healthy human subjects. The increase in the number of MVs is affected by the post - fasting concentration of total blood cholesterol and correlated with a decrease in blood glucose.

## Methods

### Blood sampling

4 mililitres of blood for measurement of blood cholesterol and glucose and 2.7 mililitres of blood for isolation of MVs (in that order) were taken by venipuncture from a medial cubital vein by a 20 gauge needle into two vacutainers (Hemogard, Beckton Dickinson, Plymouth, UK). The vacutainer for isolation of MVs contained 0.109 mol/L buffered trisodium citrate. For isolation of MVs, samples were processed within 15 minutes. Up to 8 samples were processed together, to minimize artefacts caused by keeping the samples waiting. The experiment was performed over a period of two days. The first day, blood sampling started at 7 a.m., after a 15 - hour fast by the donors. After the test, donors were encouraged to eat food rich in cholesterol, fat and carbohydrates all day. They were not restrained with respect to activities but were advised not to be extremely physically active. The second day, they consumed breakfast at 7 a.m. consisting of two eggs, bread and a dairy product and another small meal consisting of a dairy product at 10 a.m. On the second day, blood sampling from the medial cubital vein on the other arm started at 12 p.m.. For staining, blood was taken into three 4.5 mL glass vacutainers containing 0.105 mol/L sodium citrate. Vacutainers were pre - wa rmed to 37°C. Blood was drawn by a 21 gauge needle. For scanning electron microscope imaging, 30 ml of blood was allowed to freely flow into plastic containers with 1:9 v/v 0.109 mol/L buffered trisodium citrate through a 18 gauge needle (Terumo Europe, Leuwen, Belgium).

### Subjects

For determination of the concentration of MVs in blood isolates and of the concentration of cholesterol and glucose in blood, blood was taken from 33 subjects (mostly staff of the Faculty of Medicine and Faculty of Electrical Engineering, University of Ljubljana and of the University Medical Centre Ljubljana, and students of the Faculty of Medicine and of the Faculty of Veterinary Medicine, University of Ljubljana), 18 female and 15 male. Starting with 33 subjects, one male subject was excluded from the study since we found that he had undergone an 8 hour plane flight a day before taking the first blood test. Three subjects (1 male and 2 female) were excluded because we found that they did not fast for the prescribed 15 hours before the first blood test. Five subjects were excluded as their post - fasting levels of triglycerides were increased. Three subjects were excluded due to statin therapy. One subject (#11 in Table 1) received beta blocker and anti - hypertensive therapy, while 20 were medication - free. The data from two subjects on cholesterol and glucose were lost, but data on MVs of these two subjects were included in the analysis. Our facilities for blood sample processing were optimal for less than 24 samples per day, so subjects were organized into 2 groups, the first group consisting of 23 subjects and the second one consisting of 10 subjects. For antibody staining, blood was taken from a female subject (28 years) with no record of disease. For imaging with scanning electron microscope, blood was taken from a male subject (28 years) with no record of disease. All subjects gave written consent to the study which was approved by the National Ethics Committee. The study conformed to the ethical principles given in the Declaration of Helsinki.

### Determination of the number of MVs

Samples were kept in a heat bath (37°C) until being inserted into the centrifuge. According to [37], blood was centrifuged in a Centric 400 centrifuge, Tehtnica Železniki, Železniki, Slovenia at 1550 g for 15 minutes and 37°C. 250 μL of plasma were removed from the top of the vacutainer and inserted into a 1.5 mL Eppendorf tube. Samples were centrifuged at 17570 g for 30 minutes at 37°C in a Centric 400 centrifuge, Tehtnica Železniki, Železniki, Slovenia. The choice of temperature is a modification with respect to the original protocol [37] where centrifugation and isolation should be performed at room temperature. 225 μL of supernatant were removed. The pellet was resuspended in 225 μL of phosphate buffered saline containing 10.9 mmol/L trisodium citrate (PBS - citrate). Samples were vortexed for 10 seconds and centrifuged at 17570 g for 30 minutes at 37°C in the Centric 200 centrifuge (Tehtnica Železniki, Železniki, Slovenia). 75 μL of PBS - citrate were added to the pellet and vortexed for 10 seconds.

Taking the blood with vacutubes yields variations in the volumes of blood (up to ± 20%), which is important. We measured the effect of the volume of blood of the same donor on the number of isolated vesicles (with the same amount of anticoagulant in the tubes) and found a linear dependence. We corrected the number of MVs to the supposed 2.7 mL in all samples by dividing the measured number of MVs by the actual volume of blood and multiplying the result by 2.7 mL.

### Staining of MVs

Blood was centrifuged immediately after the sampling in a Hettich, Universal 320R centrifuge (Hettich LabTechnology, Germany) for 20 minutes at 1550 g and 37°C. Plasma from all 3 vacutainers was carefully collected into the same 15 mL tube, leaving the bottom 1 cm of plasma layer above the cell pellet undisturbed, and was gently mixed by turning the tube upside-down. For MV staining plasma was aliquoted (300 μL) and centrifuged at 17570 g for 30 minutes at 37°C in Sigma3K30 centrifuge (Sigma Centrifuges, UK). Upper 250 μL of the supernatant were discarded and the isolate was resuspended in PBS - citrate (250 μL) by gently vortexing. The samples were centrifuged again (17570 g, 37°C, 30 min). Upper 270 μL of the supernatant were discarded. For flow cytometric assessment of the number of MVs 70 μL of PBS - citrate was added to 30 μL of the isolate. For determination of the cellular origin of MVs the isolate (30 μL) resuspended in HEPES CaCl_2 _buffer (100 μL) was incubated for 15 minutes in the dark at the room temperature with monoclonal antibodies against platelet (mouse anti human CD42b-PE IgG1, clone HIP1, ref. No. 555473, BD Biosciences, 2.5 μL), platelet - endothelial (mouse anti human CD31-FITC IgG1, clone WM59, ref. No. 555445, BD Biosciences, 5 μL) or erythrocyte (mouse anti human CD235-FITC IgG2b, clone GA-R2, BD Biosciences, 5 μL, diluted 1:10 v/v) surface molecules or concentration-matched isotype antibodies (PE Mouse IgG1, clone MOPC-21, ref. No. 555749; FITC Mouse IgG1, clone MOPC-21, ref. No. 555748; FITC Mouse IgG2b, clone 27-35; ref. No. 555742, BD Biosciences). To remove the unbound antibodies the stained isolates were washed by the addition of 100 μl of HEPES CaCl_2 _(17570 g, 37°C, 30 minutes, Sigma 3K30).

### Flow cytometry analysis

Analysis was performed by the Altra Flow Cytometer (Beckman Coulter Inc., Fullerton, CA). Forward and side scatter (FS/SS) parameters were set at logaritmic gain. For measurement of the concentration of MVs, 20 μL of calibrating microspheres (Flow Count, Beckmann Coulter) of 10 μm size and known concentration (1.05 × 10^6^/mL) were added to the samples, diluted by an appropriate volume of PBS - citrate. At least 10000 events were recorded for each sample. The results are given by the dimensionless ratio between the number of events corresponding to MVs and the number of events corresponding to microspheres. The fluorescence intensity emitted by antibodies bound to the MV surface was detected using a 525 nm filter (PMT2) and a 610 nm filter (PMT4). For fluorescence measurements the labelled MV isolates were diluted in 180 μL of HEPES CaCl_2 _buffer. MVs were resuspended in 30 μL of the remaining supernatant and were pipetted into the flow cytometry tubes. To collect any remaining MVs, the Ependorf tubes were additionally washed with 150 μL HEPES CaCl_2_.

### Scanning electron microscopy

Blood was centrifuged at 300 g for 20 min at room temperature to obtain platelet rich plasma as supernatant. Platelet rich plasma was gathered and incubated for two hours at room temperature. MVs were isolated as described above, pelet - fixed in 1% glutaraldehyde dissolved in PBS - citrate for 60 minutes at 22 °C, post - fixed in 1% OsO_4 _dissolved in 0.9% NaCl for 60 minutes at 22°C, dehydrated in a graded series of acetone/water (50-100%, v/v) and dried in CO_2 _critical point dryer. Samples were transported to Eindhoven by air mail. For imaging, samples were coated with Pt - Pd. Imaging was performed by Quanta FEG scanning electron microscope, FEI company, Eindhoven, The Netherlands.

### Measurement of blood cholesterol and glucose

Laboratory analyses of blood cholesterol and blood glucose were performed at the Adria Lab d.o.o. Diagnostic Laboratory in Ljubljana, Slovenia. The concentrations of total cholesterol, HDL-C, LDL-C, and triglycerides in human serum were measured by enzymatic colorimetric assays on a Roche/Hitachi Cobas^® ^6000 system (Roche Diagnostic GmbH, Mannheim, Germany). Serum glucose levels were determined by an enzymatic reference method with hexokinase using the Roche/Hitachi Cobas^® ^6000 system (Roche Diagnostic GmbH, Mannheim, Germany).

### Statistical analysis

Average values, standard deviations, probabilities of statistical significance by the t - test (p) and slopes of linear dependences between parameters were calculated by Microsoft Excell software. To compare the post - fasting and post - prandial data, the paired t - test was used. Pearson coefficients (r) and the corresponding probabilities of the statistical significance of correlations (p) were calculated by SPSS software. The statistical power of differences was calculated by the PS Power and Sample Size program taking the value of α = 0.05 for type I errors.

## Competing interests

The authors declare that they have no competing interests.

## Authors' contributions

VŠ participated in the design of the study, data acquisition, statistical analysis, interpretation of results and writing the manuscript, RŠ and MF participated in the design of the study, data acquisition and writing the manuscript, AB - Z, RJ and EO participated in data acquisition, interpretation of results and critical editing of the manuscript, KM took images of isolates by scanning electron microscope and participated in critical editing of the manuscript, PV participated in the design of the study, interpretation of results and critical editing of the manuscript, VK - I participated in the design of the study, statistical analysis, interpretation of data, writing the manuscript and critical editing. All authors have read and approved the final manuscript.
